# Gastrointestinal Hemorrhage after Spontaneous Subarachnoid Hemorrhage: A Single-Center Cohort Study

**DOI:** 10.1038/s41598-017-13707-3

**Published:** 2017-10-19

**Authors:** Shang-Po Wang, Yu-Hua Huang

**Affiliations:** 1 Department of Neurosurgery, Kaohsiung Chang Gung Memorial Hospital and Chang Gung University College of Medicine, Kaohsiung, Taiwan; 2 Graduate Institute of Medicine, College of Medicine, Kaohsiung Medical University, Kaohsiung, Taiwan

## Abstract

Spontaneous subarachnoid hemorrhage (SAH) is a devastating disease, and gastrointestinal hemorrhage is one of several potential complications of acute strokes. We aim to analyze its prevalence, risk factors, and association with in-hospital prognosis following SAH. A total of 1047 adult patients with a primary diagnosis of spontaneous SAH were retrospectively enrolled. We retrieved medical information from the administrative database utilizing diagnostic and procedure codes of the International Classification of Diseases, Ninth Revision, Clinical Modification (ICD-9-CM). Patients with SAH included 418 men and 629 women, and their mean age was 57.2 (standard deviation 14.6) years (range, 18–93 years). Gastrointestinal hemorrhage occurred in 30 of the 1047 patients, accounting for 2.9%. In a multivariate logistic regression model, the independent risk factors for gastrointestinal hemorrhage were liver disease and hydrocephalus. The in-hospital mortality rates were 43.3% and 29.3% in patients with and without gastrointestinal hemorrhage, respectively, but the difference was not statistically significant. In conclusion, the prevalence of gastrointestinal hemorrhage was 2.9% in patients hospitalized for spontaneous SAH. Underlying liver disease and the presence of hydrocephalus were both independent risk factors for this complication, which is a reminder to clinicians to pay increased attention in such cases.

## Introduction

Spontaneous subarachnoid hemorrhage (SAH) is one of the catastrophic strokes with an acute fatality rate ranging from 20 to 40%^1,2^. Despite surviving the direct effects of intracranial hemorrhage, in-hospital patients usually are at high risk for multiple medical morbidities, which are significantly related to an unfavorable prognosis of SAH^3,4^. In addition, the epidemiological research reports that the prevalence of SAH increases with age, and the average age of the diseased population has risen from 52.9 to 56.6 years in recent decades^5^. Older patients are particularly susceptible to medical complications and may experience more detrimental socioeconomic consequences. As a result, it is important to determine a patient’s risk of complications to guide the level of care or clinical management decisions after SAH.

Gastrointestinal hemorrhage is a well-recognized morbidity potentially occurring during the acute phase of strokes^4,6,7^. In ischemic stroke, this complication has been studied extensively, and several risk factors have been identified^8–10^. Although gastrointestinal bleeding is relatively infrequent following cerebral ischemic events, it is associated with increased odds of death and severe dependence^6^. So far, only limited data are available to characterize acute gastrointestinal hemorrhage after SAH despite the more complicated behaviors of this stroke type.

The aim of this study was to establish the prevalence and risk factors of post-SAH gastrointestinal hemorrhage and to define whether there existed a correlation between gastrointestinal hemorrhage and short-term outcomes of SAH.

## Materials and Methods

This was a retrospective cross-sectional study carried out at Kaohsiung Chang Gung Memorial Hospital, a medical center in southern Taiwan. This research was approved by the institutional review board of Chang Gung Memorial Hospital. Since the study design was retrospective and delinked, patient informed consent was not required after approval by the institutional review board. All methods were carried out in accordance with relevant guidelines and regulations. We retrieved medical records from the administrative database, which included the following patient information: gender; age; admission and discharge dates; marital status; diagnostic codes by the International Classification of Diseases, Ninth Revision, Clinical Modification (ICD-9-CM); procedure codes; condition at discharge; and related data. From 2000 to 2010, a total of 1094 hospital admissions with a primary diagnosis of SAH (ICD-9-CM code 430) were identified. Patients who were readmitted, who were < 18 years of age, or who had missing documents were excluded. Eventually, we enrolled 1047 SAH patients for further analysis.

We investigated baseline features, including demographics and underlying diseases of hypertension (ICD-9-CM Codes 4010–4059), diabetes mellitus (ICD-9-CM Codes 2500–2509), hyperlipidemia (ICD-9-CM Codes 2720–2724), liver disease (ICD-9-CM Codes 570–573), peptic ulcer disease (ICD-9-CM Codes 53100–53491), coronary artery disease (ICD-9-Codes 4140–4149), heart failure (ICD-9-CM Codes 4280–4289), chronic pulmonary disease (ICD-9-CM Codes 490–505), chronic kidney disease (ICD-9-CM Codes 585–586), coagulopathy (ICD-9-CM Codes 2860–2869), and thrombocytopenia (ICD-9-CM Codes 2870–2875).

Major therapeutic interventions were recorded; these included surgical treatments for cerebral aneurysms (Procedure Codes 3951–3952), endovascular interventions for cerebral aneurysms (Procedure Code 3979), mechanical ventilation for 96 hours or longer (Procedure Code 9672), and tracheostomy procedures (Procedure Codes 311, 3121, or 3129).

Patients suffering from gastrointestinal hemorrhage were recognized when coded as ICD-9-CM 5780–5789. Other medical complications included diabetes insipidus (ICD-9-CM Code 2535), hypernatremia or hyperosmolarity (ICD-9-CM Code 2760), hyponatremia or hypoosmolarity (ICD-9-CM Code 2761), hyperpotassemia (ICD-9-CM Code 2767), hypopotassemia (ICD-9-CM Code 2768), anemia (ICD-9-CM Codes 2851 & 2859), acute kidney failure (ICD-9-CM Codes 5845–5849), pneumonia (ICD-9-CM Codes 481–486), or urinary tract infection (ICD-9-CM Code 5990). Neurological complications included central nervous system infection (ICD-9-CM Codes 3200–3249), hydrocephalus (ICD-9-CM Codes 3313–3314), cerebral ischemia or infarction (ICD-9-CM Codes 4330–4371), convulsion (ICD-9-CM Code 7803), or hemiplegia (ICD-9-CM Codes 3420–3429). The short-term outcomes focused on in this study were in-hospital mortality rate and length of hospital stay.

We analyzed data with SPSS software (IBM SPSS Statistics, version 20.0). Parameters were presented as numbers (percentage) or as a mean ± standard deviation (SD). Intergroup differences were assessed using the chi-square test or Fisher’s exact test for categorical variables, and the Student’s *t*-test or Mann-Whitney *U*-test for continuous variables. All parameters with a *P* value < 0.1 were included in multivariable logistic regression to adjust for independent risk factors of gastrointestinal hemorrhage after SAH. A Kaplan–Meier survival curve was constructed and compared using the log-rank test. Statistical significance was defined as a *p* < 0.05.

## Results

The 1047 patients, including 418 men and 629 women, were diagnosed with first-ever spontaneous SAH. The mean age was 57.2 ± 14.6 years (range, 18–93 years) at the time of diagnosis. The median of length of hospital stay was 12 days (range, 1–102 days). Of these patients, 353 (33.7%) underwent surgical treatments and 172 (16.4%) underwent endovascular interventions for cerebral aneurysms. There were 256 (24.5%) cases who had mechanical ventilation for 96 hours or longer and 61 (5.8%) received tracheostomy procedures.

**Figure 1:**
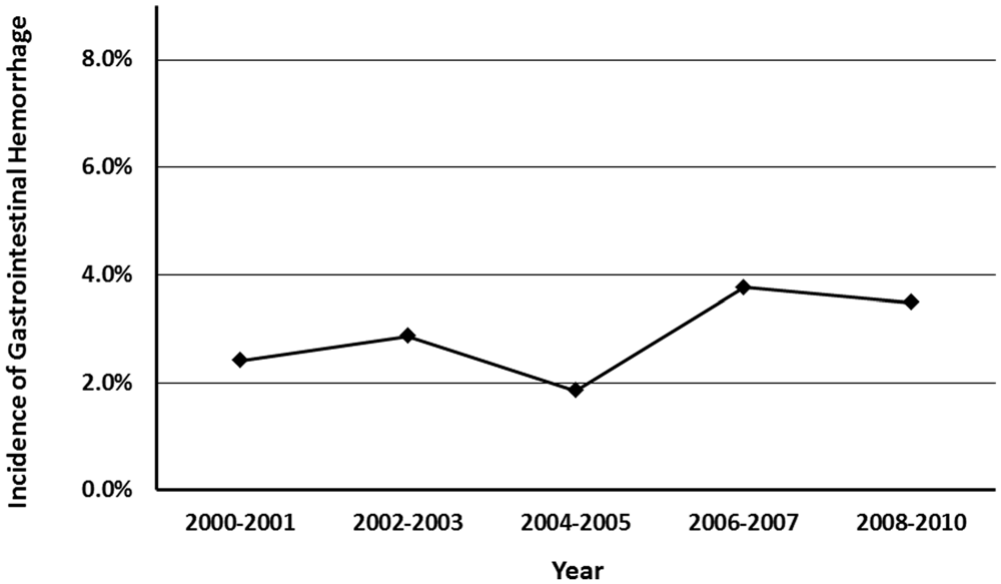
Prevalence of gastrointestinal hemorrhage after spontaneous SAH over time.

Gastrointestinal hemorrhage was documented in 30 of the 1047 patients hospitalized for SAH, and the overall prevalence was 2.9%. From 2000 to 2010, the occurrence of gastrointestinal hemorrhage did not change obviously, and the prevalence was maintained between 2% and 4% over time (Fig. 1). The other in-hospital morbidities were as follows: 9 (0.9%) diabetes insipidus, 18 (1.7%) hypernatremia or hyperosmolarity, 41 (3.9%) hyponatremia or hypoosmolarity, 6 (0.6%) hyperpotassemia, 80 (7.6%) hypopotassemia, 99 (9.5%) anemia, 10 (1.0%) acute kidney failure, 111 (10.6%) pneumonia, 164 (15.7%) urinary tract infection, 41 (3.9%) central nervous system infection, 352 (33.6%) hydrocephalus, 100 (9.6%) cerebral ischemia or infarction, 54 (5.2%) convulsion, and 56 (5.3%) hemiplegia.

In a comparison of clinical characteristics of patients with or without gastrointestinal hemorrhage, statistical analysis identified the following parameters with a *P* value < 0.10: gender (*p* = 0.06), liver disease (*p* = 0.01), thrombocytopenia (*p* = 0.05), pneumonia (*p* = 0.03), hydrocephalus (*p* < 0.01), and mechanical ventilation for 96 hours or longer (*p* < 0.01) (Table 1). All these factors were included in multivariable regression analysis, and the independent risk factors for gastrointestinal hemorrhage following SAH included underlying liver disease (*p* = 0.03) and hydrocephalus (*p* < 0.01) (Table 2).

**Table 1:** Comparisons of clinical features between patients with or without gastrointestinal hemorrhage after SAH.

		Gastrointestinal hemorrhage		p
	Total cases	Yes	No	
	N = 1047	N = 30	N = 1017	
	n(%)	n(%)	n(%)	
Gender				0.06
Male	418 (39.9)	17 (56.7)	401 (39.4)	
Female	629 (60.1)	13 (43.3)	616 (60.6)	
Age (years)				0.77
<40	122 (11.7)	5 (16.7)	117 (11.5)	
40–49	211 (20.2)	7 (23.3)	204 (20.1)	
50–59	250 (23.9)	6 (20.0)	244 (24.0)	
≥60	464 (44.3)	12 (40.0)	452 (44.4)	
Marital status				1.00
Married	991 (94.7)	29 (96.7)	962 (94.6)	
Single	56 (5.3)	1 (3.3)	55 (5.4)	
Onset of seasonal stratification				0.60
Spring	264 (25.2)	9 (30.0)	255 (25.1)	
Summer	238 (22.7)	8 (26.7)	230 (22.6)	
Fall	257 (24.5)	8 (26.7)	249 (24.5)	
Winter	288 (27.5)	5 (16.7)	283 (27.8)	
Underlying diseases				
Hypertension	461 (44.0)	15 (50.0)	446 (43.9)	0.50
Diabetes mellitus	101 (9.6)	2 (6.7)	99 (9.7)	0.76
Hyperlipidemia	14 (1.3)	0 (0.0)	14 (1.4)	1.00
Liver disease	33 (3.2)	4 (13.3)	29 (2.9)	0.01
Peptic ulcer	16 (1.5)	0 (0.0)	16 (1.6)	1.00
Coronary artery disease	11 (1.1)	0 (0.0)	11 (1.1)	1.00
Heart failure	9 (0.9)	0 (0.0)	9 (0.9)	1.00
Chronic pulmonary disease	17 (1.6)	1 (3.3)	16 (1.6)	0.39
Chronic kidney disease	13 (1.2)	0 (0.0)	13 (1.3)	1.00
Coagulopathy	8 (0.8)	1 (3.3)	7 (0.7)	0.21
Thrombocytopenia	13 (1.2)	2 (6.7)	11 (1.1)	0.05
In-hospital complications				
Diabetes insipidus	9 (0.9)	1 (3.3)	8 (0.8)	0.23
Hypernatremia / Hyperosmolarity	18 (1.7)	0 (0)	18 (1.8)	1.00
Hyponatremia / Hyposmolarity	41 (3.9)	2 (6.7)	39 (3.8)	0.33
Hyperpotassemia	6 (0.6)	1 (3.3)	5 (0.5)	0.16
Hypopotassemia	80 (7.6)	3 (10.0)	77 (7.6)	0.50
Anemia	99 (9.5)	4 (13.3)	95 (9.3)	0.52
Acute kidney failure	10 (1.0)	1 (3.3)	9 (0.9)	0.25
Pneumonia	111 (10.6)	7 (23.3)	104 (10.2)	0.03
Urinary tract infection	164 (15.7)	6 (20.0)	158 (15.5)	0.45
Central nervous system infection	41 (3.9)	2 (6.7)	39 (3.8)	0.33
Hydrocephalus	352 (33.6)	18 (60.0)	334 (32.8)	< 0.01
Cerebral ischemia / infarction	100 (9.6)	3 (10.0)	97 (9.5)	0.76
Convulsion	54 (5.2)	1 (3.3)	53 (5.2)	1.00
Hemiplegia	56 (5.3)	3 (10.0)	53 (5.2)	0.21
In-hospital managements				
Surgical procedure for aneurysm	353 (33.7)	9 (30.0)	344 (33.8)	0.66
Endovascular procedure for aneurysm	172 (16.4)	3 (10.0)	169 (16.6)	0.46
Mechanical ventilation ≥ 96hr	256 (24.5)	15 (50.0)	241 (23.7)	< 0.01
Tracheostomy	61 (5.8)	0 (0.0)	61 (6.0)	0.41

**Table 2:** Multivariable analysis of independent risk factors for gastrointestinal hemorrhage after SAH.

	Gastrointestinal hemorrhage	
	Odds ratio (95% CI)	P value
Gender	1.91 (0.90–4.07)	0.09
Liver disease	4.40 (1.17–16.52)	0.03
Thrombocytopenia	4.98 (0.79–31.37)	0.09
Pneumonia	1.40 (0.52–3.74)	0.51
Hydrocephalus	3.08 (1.35–7.02)	< 0.01
Mechanical ventilation ≥ 96hr	2.27 (0.96–5.36)	0.06

**Figure 2:**
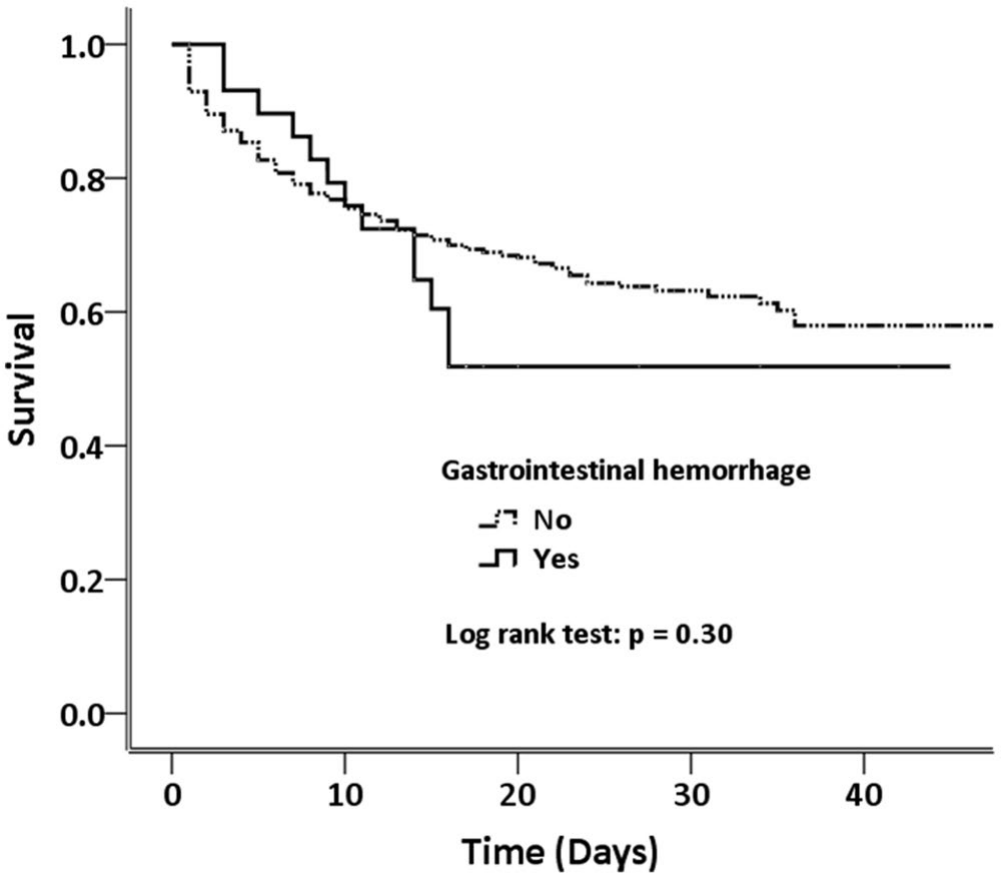
Kaplan–Meier survival curves for SAH patients with or without gastrointestinal hemorrhage.

The mean duration of hospital stay was 15.7 ± 10.1 and 14.5 ± 12.8 days for patients with and without gastrointestinal hemorrhage, respectively (p = 0.21). At discharge, 13 of the 30 patients with gastrointestinal hemorrhage and 298 of the 1017 patients without gastrointestinal hemorrhage had died; thus, incidences of in-hospital mortality were 43.3% and 29.3%, respectively (*p* = 0.10). Kaplan–Meier survival curves for the two populations with spontaneous SAH showed that, by the 30th day of hospitalization, 56.7% of the gastrointestinal hemorrhage group and 71.2% of the non-gastrointestinal-hemorrhage group had survived (Fig. 2). No remarkable difference was observed between the two study groups (log-rank test: *p* = 0.30).

## Discussion

SAH accounts for less than 10% of all strokes, with an overall prevalence of approximately 9 per 100,000 person-years^11,12^. However, as a neurological emergency with a serious outcome impact, SAH remains an intensive topic of clinical studies, and the characteristics of the complications are generally investigated^3,4,12^. Our study showed that gastrointestinal hemorrhage occurred in 2.9% of the 1047 adults with SAH during acute hospitalization. In a study focusing on post-SAH medical morbidities, Wartenberg *et al*. reported a gastrointestinal bleeding rate of 4% among 576 patients, which was similar to that observed by us^3^. In comparison, the prevalence of gastrointestinal hemorrhage was 3% after acute strokes (excluding SAH)^7^, and only 1.2%–1.5% in patients with ischemic stroke^6,8,9^. Therefore, though gastrointestinal hemorrhage following SAH is uncommon, its frequency is still higher than that of ischemic cerebral events which usually require subsequent thromboprophylaxis.

The pathophysiology of gastrointestinal hemorrhage after acute strokes is not completely realized, and several theories exist. Intracranial lesions or elevated intracranial pressure affect brainstem or hypothalamic nuclei with hyperactivity of vagal tone and lead to hemorrhagic peptic ulcerations because of hypersecretion of gastric acid^13^. Second, acute stroke predisposes to gastroparesis resulting from interruption of the axis between the central nervous and digestive systems and seems to increase the risk of gastrointestinal bleeding^14^. In addition, a humoral surge in catecholamines or cortisol is usual in the acute phase of stroke^15^ and possibly causes vasoconstriction or mucosal damage of the gastrointestinal tract. As we all know, SAH is a highly stressful disease, and it is reported that intracranial hypertension is common after SAH, even in 48.7% of good-grade cases^16^. This also explains why the occurrence of gastrointestinal hemorrhage is more frequent in patients with SAH than in those with ischemic stroke.

Many risk factors of gastrointestinal hemorrhage after acute ischemic stroke, such as previous history of peptic ulcer disease, liver disease, cancer, sepsis, renal insufficiency, or stroke severity, have been documented^6,8–10^. In patients suffering from non-traumatic intracerebral hemorrhage, increased hematoma size, septicemia, and low Glasgow coma scale score are at risk for gastrointestinal hemorrhage^17^. Our results demonstrated that underlying liver disease and the presence of hydrocephalus were both independent risk factors for post-SAH gastrointestinal hemorrhage. In contrast to ischemic stroke, it is surprising that our patients with previous peptic ulcers were not vulnerable to subsequent gastrointestinal bleeding. The reasonable explanation is that the comorbidities were probably underestimated, as ICD-9-CM code recording is occasionally incomplete in clinical practice. Additionally, the number of patients may still be relatively small from a statistical standpoint, and this is underpowered to detect the significance of some risk factors.

A connection between liver disease and gastrointestinal hemorrhage is noted in populations with either ischemic stroke or SAH. Rumalla *et al*. analyzed acute ischemic stroke in the largest database of the United States, but information on the subgroup with liver disease is scanty^9^. Interestingly, a study from Taiwan reported that abnormal liver function is related to gastrointestinal hemorrhage after ischemic stroke^10^, which is compatible with our findings. Taiwan is one of the endemic areas for hepatitis B and hepatitis C viral infections, and the estimated prevalence rates are 13.70% and 1.8%–5.5%, respectively^18,19^. In this series, the overall prevalence of liver disease is merely 3.2%, and appears to be understated, perhaps owing to the absence of personal awareness or general surveillance programs for hepatitis infections. Theoretically, patients with chronic liver diseases have gastrointestinal bleeding most commonly from gastroesophageal varices or gastropathy associated with portal hypertension, but non-variceal bleeding is not infrequent^20^. Clinicians must pay attention to liver function and thrombocoagulation status after SAH, especially when patients have potential liver diseases with unpredictable sources or pathogenesis of hemorrhagic gastrointestinal tract lesions.

The occurrence of hydrocephalus is a critical complication following SAH, and there are several predictive factors, including high Hunt and Hess Scale score, high Fisher score, rehemorrhage, presence of intraventricular blood, posterior circulation aneurysm location, or older age^21,22^. Most of these predictors for hydrocephalus are correlated to initial clinical severity or subarachnoid blood volume. With a greater amount of hemorrhage, the subarachnoid space is filled with blood cells and corresponding products which block the outflow tract of cerebrospinal fluid, and thus the presence of hydrocephalus can be viewed as a sign of severe SAH. Moreover, acute cerebrospinal fluid disarrangement is likely accompanied by a rise in intracranial pressure. Hydrocephalus with subsequent intracranial pressure increase potentially induces a surge of stress hormones, and may explain why hydrocephalus is significantly relevant to post-SAH gastrointestinal hemorrhage.

In this study, gastrointestinal hemorrhage was not a remarkable factor correlated with the prognosis of SAH. Nevertheless, there seemed to be a trend in the difference between the in-hospital mortality rates in patients with and without gastrointestinal hemorrhage (43.3% and 29.3%, respectively). In a total of 6853 patients with acute ischemic stroke, O’Donnell *et al*. showed that gastrointestinal hemorrhage was strongly associated with in-hospital death and 6-month mortality^6^. Another population-based study for ischemic stroke similarly reported an 82% increased likelihood of death at discharge in patients with gastrointestinal bleeding^9^. Because our findings reflect the observation of a single medical center, we suppose that the current evidence may be insufficient. Further studies in a larger series of cases are required to draw a firm conclusion on the relationship between gastrointestinal hemorrhage and post-SAH outcomes.

There were several limitations to this study. First, the utility of ICD-9-CM coding for research was sometimes questionable, because the specific information, such as Hunt and Hess grading, Fisher grading, location or size of cerebral aneurysms, or sequential functional status, was difficult to evaluate. Second, the analysis did not contain records regarding the use of proton pump inhibitors or histamine receptor antagonists, which have a potential impact on the occurrence or severity of gastrointestinal hemorrhage. Third, the etiology of gastrointestinal bleeding was not documented, though we considered that bleeding originating from the upper gastrointestinal tract accounted for the majority of cases. Fourth, the outpatient or longer-term prognosis of SAH was not addressed here, and this is an important consideration from a public health perspective. Fifth, because of the higher rate of hepatitis in our country, these results may not be generalized to the non-endemic area. In addition, we enrolled the patients with a primary diagnosis of SAH (ICD-9-CM code 430), and this cohort included 15% non-aneurysmal SAH^23^. Since SAH is a disease with around 30% early mortality rate^1,2^, the patients with poor grading SAH may have no chance to undergo cerebral angiography or further managements. These explained why only about 50% of our SAH-patients received definite treatments of aneurysms.

In conclusions, the prevalence of gastrointestinal hemorrhage was 2.9% in patients hospitalized for spontaneous SAH. Underlying liver disease and the presence of hydrocephalus were both independent risk factors for this complication, which reminds clinicians to pay increased attention in such cases.
